# Die Verordnung (EU) 2017/746 (IVDR) in der Praxis: Umsetzung von Anhang I in der Pathologie

**DOI:** 10.1007/s00292-023-01231-3

**Published:** 2023-10-04

**Authors:** Andy Kahles, Hannah Goldschmid, Anna-Lena Volckmar, Carolin Ploeger, Daniel Kazdal, Roland Penzel, Jan Budczies, Christa Flechtenmacher, Ulrich M. Gassner, Monika Brüggemann, Michael Vogeser, Peter Schirmacher, Albrecht Stenzinger

**Affiliations:** 1 Pathologisches Institut, Universitätsklinikum Heidelberg, Im Neuenheimer Feld 224, 69120 Heidelberg, Deutschland; 2 Juristische Fakultät, Universität Augsburg, Augsburg, Deutschland; 3 Klinik für Innere Medizin II, Sektion für Hämatologische Spezialdiagnostik, Universitätsklinikum Schleswig-Holstein, Kiel, Deutschland; 4 Labormedizin, Klinische Massenspektrometrie, LMU München, München, Deutschland

**Keywords:** Qualitätsmanagement, Qualitätssicherung in der Gesundheitsversorgung, Regulatorische Anforderungen, Eigenherstellung, Laboratory-developed-Test, Quality Management, Quality Assurance in health care, Regulatory requirements, In-house manufacturing, Laboratory-developed tests

## Abstract

**Hintergrund:**

Die Verordnung (EU) 2017/746 über *In-vitro-*Diagnostika (IVDR) stellt mehrere Bedingungen an Pathologische Institute, die hausinterne *In-vitro-*Diagnostika (IH-IVD) entwickeln und anwenden. Diese Bedingungen müssen jedoch nicht alle unmittelbar mit dem Geltungsbeginn der IVDR zum 26.05.2022 umgesetzt worden sein. Auf der Grundlage einer Änderungsverordnung des Europäischen Parlaments und des Rates der Europäischen Union werden die Anforderungen an IH-IVD stufenweise eingeführt. Die Konformität mit den grundlegenden Sicherheits- und Leistungsanforderungen gemäß Anhang I muss seit Mai 2022 gewährleistet sein

**Ziel der Arbeit:**

Mit diesem Artikel möchten wir die praktische Umsetzung der aktuell gültigen Bedingungen für IH-IVD im Pathologischen Institut des Universitätsklinikums Heidelberg vorstellen und damit mögliche Hilfestellung für andere Einrichtungen geben

**Schlussfolgerungen:**

Neben der intensiven Auseinandersetzung mit den Anforderungen an IH-IVD geben mehrere Handreichungen und Hilfestellungen eine Orientierungshilfe zur Umsetzung und Harmonisierung der in Artikel 5 (5) genannten Anforderungen an Gesundheitseinrichtungen. Auch der Austausch in akademischen Netzwerkstrukturen ist für die Interpretation und die praktische Umsetzung der IVDR von großer Bedeutung. Für universitäre und nicht-universitäre Einrichtungen stellt die Sicherstellung der IVDR-Konformität – neben den wesentlichen Aufgaben in der Krankenversorgung, in der Lehre und der Forschung und Weiterentwicklung von Methoden zur optimalen und zielgerichteten Diagnostik sowie der Aufrechterhaltung des sich stetig weiterentwickelnden Qualitätsmanagementsystems  – eine weitere personelle und zeitliche Herausforderung dar.

## Abstract (en)

**Background:**

Regulation (EU) 2017/746 on in vitro diagnostic medical devices (IVDR) imposes several conditions on pathology institutes that develop and use in-house in vitro diagnostic medical devices (IH-IVDs). However, not all of these conditions need to be implemented immediately after the IVDR entered into force on 26 May 2022. Based on an amending regulation of the European Parliament and the Council of the European Union, the requirements for IH-IVDs will be phased in. Conformity with the essential safety and performance requirements of annex I must be ensured from May 2022.

**Objectives:**

With this article, we would like to present the practical implementation of the currently valid conditions for IH-IVDs at the Institute of Pathology at the University Hospital of Heidelberg, in order to provide possible assistance to other institutions.

**Conclusions:**

In addition to the intensive work on the requirements for IH-IVDs, several guidance documents and handouts provide orientation for the implementation and harmonisation of the requirements for healthcare institutions mentioned in Article 5 (5). Exchange in academic network structures is also of great importance for the interpretation and practical implementation of the IVDR. For university and nonuniversity institutions, ensuring conformity with the IVDR represents a further challenge in terms of personnel and time, in addition to the essential tasks of patient care, teaching and research and the further development of methods for optimal and targeted diagnostics, as well as the maintenance of the constantly evolving quality management system.

## Einleitung

Die „Verordnung (EU) 2017/746 des Europäischen Parlaments und des Rates vom 5. April 2017 über *In-vitro-*Diagnostika und zur Aufhebung der Richtlinie 98/79/EG und des Beschlusses 2010/227/EU der Kommission“ ([16]; IVDR) ist am 26. Mai 2017 in Kraft getreten und gilt seit dem 26. Mai 2022. Das Kernziel der EU-weit geltenden Verordnung ist es, durch harmonisierte Anforderungen an die Herstellung und Anwendung von *In-vitro-*Diagnostika (IVD) ein höchstmögliches Maß an Gesundheitsschutz für Patientinnen und Patienten in Verbindung mit einer hohen Anwendersicherheit zu gewährleisten.

In diesem Artikel beschreiben wir die Vorgehensweise bei der Implementierung der aktuell geltenden Anforderungen der IVDR für Gesundheitseinrichtungen und der „Grundlegenden Sicherheits- und Leistungsanforderungen“ (IVDR, Anhang I) in das etablierte Qualitätsmanagement-(QM)-system des Pathologischen Instituts der Universität Heidelberg (IPH).

## Die IVDR: Anforderungen an Pathologien

Die IVDR unterscheidet zwei Arten von IVDR-konformen IVD (Tab. 1): CE-gekennzeichnete IVD (CE-IVD) von Wirtschaftsakteuren und In-house-IVD (IH-IVD) von Gesundheitseinrichtungen (Begriffsdefinitionen siehe Tab. 2). Für die komplexe Diagnostik in der Pathologie werden beide IVD-Varianten – auch kombiniert miteinander – eingesetzt, um eine optimale Patientenversorgung zu gewährleisten.

**Table 1:** 

CE-IVD	IH-IVD
*Hersteller: *Wirtschaftsakteure *Bereitstellung:* Europäischer Markt	*Hersteller: *Gesundheitseinrichtungen (z. B. Pathologie) *Bereitstellung:* innerhalb der Herstellungsinstitution
*Alle* Anforderungen der IVDR sind zu erfüllen; abhängig von der Art und der Risikoklasse des Produkts	*Artikel 5 (5***) **ist zu erfüllen, inkl. Anhang I
Benannte Stellen sind für die Konformitätsbewertung mit der IVDR zuständig: Aktuell 10 × in der EU (Stand 05/2023 [6])	Ohne Beteiligung von Benannten Stellen; Überwachung durch zuständige Landesbehörde
Registrierung der Produkte in der Europäischen Datenbank für Medizinprodukte (EUDAMED; [5])	Keine EUDAMED-Registrierung

In Erwägungsgrund 29 der IVDR wird die besondere Bedeutung von Gesundheitseinrichtungen und den von ihnen selbst entwickelten IVD beschrieben. Danach sollen Gesundheitseinrichtungen – und damit auch die Pathologischen Institute und Zentren – weiterhin die Möglichkeit haben, Produkte hausintern herzustellen, zu verändern und zu verwenden, um damit auf die spezifischen Bedürfnisse der Patientenzielgruppen eingehen zu können. Hierfür soll die IVDR jedoch EU-weit harmonisierte Vorschriften vorgeben (Erwägungsgrund 28), die in Artikel 5, Absatz 5 beschrieben werden.

**Figure 1:**
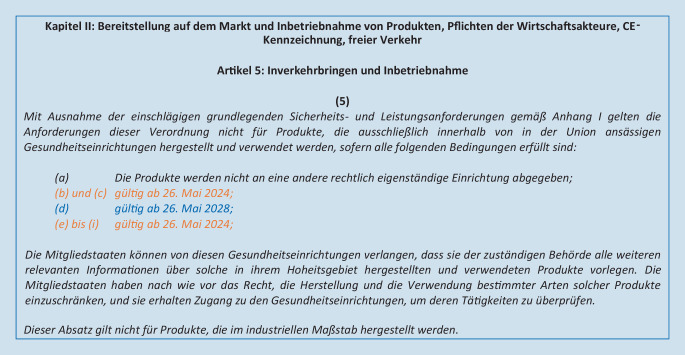

Die IVDR legt in diesem Artikel mehrere Bedingungen für Gesundheitseinrichtungen fest, die IH-IVD entwickeln und verwenden. Sofern alle darin beschriebenen Bedingungen erfüllt sind, gelten sämtliche darüberhinausgehenden Anforderungen der IVDR nicht für IH-IVD. Diese Bedingungen für Gesundheitseinrichtungen treten nach einer Änderungsverordnung vom Januar 2022 [17] stufenweise in Kraft (Abb. 1).

Die nachfolgend zusammengefassten Anforderungen müssen bereits seit 26. Mai 2022 beachtet werden:

1. IH-IVD müssen den grundlegenden Sicherheits- und Leistungsanforderungen entsprechen (IVDR, Anhang I).
2. Die Erstellung und die Verwendung müssen innerhalb der EU geschehen.
3. IH-IVD dürfen nur von der Institution selbst verwendet und nicht abgegeben werden.
4. Die zuständigen Behörden (auf Landesebene) erhalten auf Anfrage einschlägige Informationen über die Produkte und erhalten Zugang zu den Gesundheitseinrichtungen, um deren Tätigkeiten zu überprüfen.
5. IH-IVD dürfen nicht in einem industriellen Maßstab produziert werden.

Die Bezeichnung „industrieller Maßstab“ ist in der IVDR nicht definiert. Jedoch nimmt das Leitliniendokument MDCG-2023‑1, das speziell für IH-IVD von der Koordinierungsgruppe Medizinprodukte (MDCG, *Medical Device Coordination Group*) erstellt und im Januar 2023 veröffentlicht wurde, dazu Stellung [13]. Diese Guideline führt hierzu aus, dass im Herstellungsprozess nicht mehr als die geschätzte Anzahl der benötigten Produkte hergestellt werden sollte.

Für die Punkte (2–5) ergibt sich somit kein unmittelbarer Handlungsbedarf für die Pathologien. Jedoch müssen sich Pathologien mit Punkt (1), den „grundlegenden Sicherheits- und Leistungsanforderungen“, auseinandersetzen, die in Anhang I der IVDR beschrieben sind.

**Table 2:** 

Begriff	Begriffsbestimmung laut Verordnung (EU) 2017/746 (IVDR) [16]	Verweis
Analyseleistung	„Analyseleistung bezeichnet die Fähigkeit eines Produkts, einen bestimmten Analyten korrekt nachzuweisen oder zu messen.“	Artikel 2 (40)
Anwender	„Anwender bezeichnet jeden Angehörigen der Gesundheitsberufe oder Laien, der ein Produkt anwendet.“	Artikel 2 (30)
Gebrauchsanweisung	„Gebrauchsanweisung bezeichnet vom Hersteller zur Verfügung gestellte Informationen, in denen der Anwender über die Zweckbestimmung und korrekte Verwendung eines Produkts sowie über eventuell zu ergreifende Vorsichtsmaßnahmen unterrichtet wird.“	Artikel 2 (14)
Gesundheitseinrichtung	„Gesundheitseinrichtung bezeichnet eine Organisation, deren Hauptzweck in der Versorgung oder Behandlung von Patienten oder der Förderung der öffentlichen Gesundheit besteht.“	Artikel 2 (29)
Hersteller	„Hersteller bezeichnet eine natürliche oder juristische Person, die ein Produkt herstellt oder als neu aufbereitet bzw. entwickeln, herstellen oder als neu aufbereiten lässt und dieses Produkt unter ihrem eigenen Namen oder ihrer eigenen Marke vermarktet.“	Artikel 2 (23)
Kennzeichnung	„Kennzeichnung bezeichnet geschriebene, gedruckte oder grafisch dargestellte Informationen, die entweder auf dem Produkt selbst oder auf der Verpackung jeder Einheit oder auf der Verpackung mehrerer Produkte angebracht sind.“	Artikel 2 (13)
Klinische Leistung	„klinische Leistung bezeichnet die Fähigkeit eines Produkts, Ergebnisse zu liefern, die mit einem bestimmten klinischen Zustand oder physiologischen oder pathologischen Vorgang oder Zustand bei einer bestimmten Zielbevölkerung und bestimmten vorgesehenen Anwendern korrelieren.“	Artikel 2 (41)
Leistung eines Produkts	„Leistung eines Produkts bezeichnet die Fähigkeit eines Produkts, seine vom Hersteller angegebene Zweckbestimmung zu erfüllen; sie besteht in der Analyseleistung und gegebenenfalls der klinischen Leistung zur Erfüllung dieser Zweckbestimmung.“	Artikel 2 (39)
Leistungsbewertung	„Leistungsbewertung bezeichnet eine Beurteilung und Analyse von Daten zur Feststellung oder Überprüfung der wissenschaftlichen Validität, der Analyseleistung und gegebenenfalls der klinischen Leistung eines Produkts.“	Artikel 2 (44)
Nutzen-Risiko-Abwägung	„Nutzen-Risiko-Abwägung bezeichnet die Analyse aller Bewertungen des Nutzens und der Risiken, die für die bestimmungsgemäße Verwendung eines Produkts entsprechend der vom Hersteller angegebenen Zweckbestimmung von möglicher Relevanz sind.“	Artikel 2 (17)
Risiko	„Risiko bezeichnet die Kombination von Wahrscheinlichkeit eines Schadenseintritts und Schwere des Schadens.“	Artikel 2 (16)
Wirtschaftsakteur	„Wirtschaftsakteur bezeichnet einen Hersteller, einen Bevollmächtigten, einen Importeur oder einen Händler.“	Artikel 2 (28)
Zweckbestimmung	„Zweckbestimmung bezeichnet die Verwendung, für die ein Produkt entsprechend den Angaben des Herstellers auf der Kennzeichnung, in der Gebrauchsanweisung oder dem Werbe- oder Verkaufsmaterial bzw. den Werbe- oder Verkaufsangaben oder seinen Angaben bei der Leistungsbewertung bestimmt ist.“	Artikel 2 (12)

## Umsetzung von Anhang I in der Pathologie

Pathologien in Deutschland werden durch die Deutsche Akkreditierungsstelle GmbH (DAkkS) nach DIN EN ISO/IEC 17020 als Inspektionsstellen akkreditiert. Im Mittelpunkt der Akkreditierung steht die sachverständige Beurteilung der Pathologin, bzw. des Pathologen und damit die Bestätigung der fachlichen Kompetenz der Inspektionsstelle [7]. Die Anforderungen der Norm EN ISO 15189 müssen dabei ebenfalls berücksichtigt werden [4]. Die Abteilung Allgemeine Pathologie und pathologische Anatomie des Pathologischen Institutes am Universitätsklinikum Heidelberg (IPH) ist seit 2007 akkreditiert. Damit steht eine etablierte, solide und unabhängig geprüfte Qualitätsmanagementstruktur zur Verfügung, in die die Anforderungen der IVDR integriert werden können. Die IVDR fordert in Artikel 5 (5) c) ein Qualitätsmanagementsystem entsprechend der Norm EN ISO 15189, schreibt jedoch keine Akkreditierung vor [8].

### Anhang I: grundlegende Sicherheits- und Leistungsanforderungen

**Figure 2:**
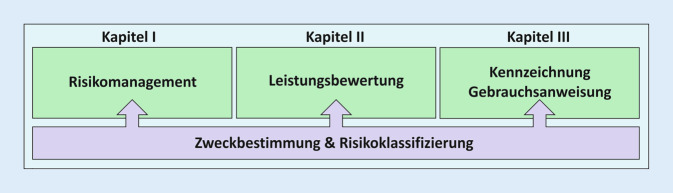

Alle IVD-Produkte – unabhängig davon, ob es sich um kommerzielle CE-IVD oder IH-IVD aus Eigenherstellung handelt – müssen die Sicherheits- und Leistungsanforderungen erfüllen (Artikel 5 [5]). Mit diesen Anforderungen soll sichergestellt und nachgewiesen werden, dass die Produkte für Patient/innen und Anwender/innen sicher sind, dass mögliche Risiken bekannt und kontrolliert sind und dass die Produkte für ihren Verwendungszweck geeignet sind. Als Hersteller und Anwender von IH-IVD muss die Pathologie die Konformität mit Anhang I sicherstellen und nachweisen. Anhang I ist in drei Kapitel unterteilt (Abb. 2):

- Kapitel I: Allgemeine Anforderungen (→Fokus auf Risikomanagement),
- Kapitel II: Anforderungen an Leistung, Auslegung und Herstellung (→Fokus auf Leistungsbewertung),
- Kapitel III: Anforderungen an die mit dem Produkt gelieferten Informationen (→Fokus auf Kennzeichnung und Gebrauchsanweisung).

Die Zweckbestimmung bildet die Grundlage für die Anforderungen aus Anhang I. Sie bestimmt damit den Aufwand für die Anforderungen der Kapitel I, II und III und den Umfang der zugehörigen Dokumentation.

### Zweckbestimmung & Risikoklassifizierung

Die Festlegung der Zweckbestimmung erhöht die Sicherheit von Patient/innen und Anwender/innen, da eine nicht bestimmungsgemäße Verwendung des Produkts unwahrscheinlicher wird und eine mögliche Zweckentfremdung vermieden wird. Darüber hinaus basieren die „Grundlegenden Sicherheits- und Leistungsanforderungen“ in Anhang I auf der produktspezifischen Zweckbestimmung. Die Zweckbestimmung hat daher einen entscheidenden Einfluss auf den Arbeitsaufwand für die drei zentralen Themen in Anhang I (Abb. 2). „Zweckbestimmung bezeichnet die Verwendung, für die ein Produkt entsprechend den Angaben des Herstellers auf der Kennzeichnung, in der Gebrauchsanweisung […] oder seinen Angaben bei der Leistungsbewertung bestimmt ist“ (ungekürzte Definition siehe Tab. 2).

Die Zweckbestimmung ist ausschlaggebend für die Klassifizierung des Produkts. Die IVDR beschreibt sieben Regeln, um IVD, entsprechend ihrer Zweckbestimmung, in vier Klassen A, B, C und D mit steigendem individuellem und öffentlichem Risiko zu klassifizieren (Anhang VIII). Je fataler die Folgen einer möglichen Fehldiagnose für den Patienten/die Patientin sind (z. B. bei lebensbedrohlichen Erkrankungen) und je größer die Gefahr für die Bevölkerung ist (z. B. bei übertragbaren Erregern lebensbedrohlicher Krankheit), desto höher ist die Risikoklasse.

**Figure 3:**
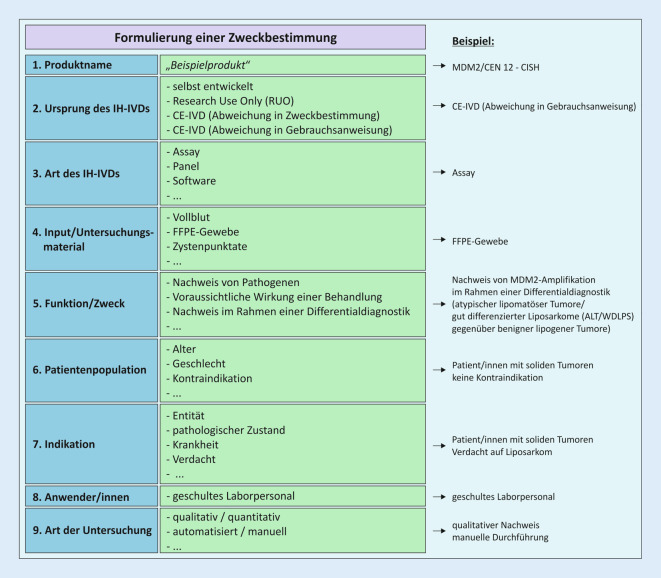

Für eine einheitliche und damit vergleichbare produktspezifische Formulierung der Zweckbestimmung hat das IPH eine neue Arbeitsanweisung in die bestehende QM-Dokumentation implementiert. Die Arbeitsanweisung beschreibt, wie die Zweckbestimmung für intern entwickelte Untersuchungsverfahren (IH-IVD) formuliert wird und wie die Risikoklassifizierung durchzuführen ist. Dabei werden mehrere Komponenten (Produktname, die Art des IH-IVD, das Untersuchungsmaterial, Funktion bzw. Zweck, Patientenpopulation, Indikation, Anwenderkreis und Anwendungsbereich) produktspezifisch definiert (Abb. 3). Alle diese Komponenten werden in einem Formular in Form einer Checkliste abgefragt und damit die Zweckbestimmung festgelegt. Anschließend erfolgt auf demselben Formblatt die Risikoklassifizierung gemäß Anhang VIII der IVDR. Eine Hilfestellung hierzu bietet auch das Leitliniendokument MDCG-2020-16 der Koordinierungsgruppe Medizinprodukte [12].

### Anhang I, Kapitel I: allgemeine Anforderungen

Kapitel I fokussiert auf das produktspezifische Risikomanagement für die Herstellung und Anwendung von IH-IVD. IH-IVD müssen geeignet und sicher sein; sie dürfen weder die Sicherheit der Patient/innen noch die Sicherheit und die Gesundheit der Anwender/innen oder gegebenenfalls Dritter gefährden. Ein mögliches Risiko, das mit ihrer Herstellung und Anwendung verbunden ist, muss im Verhältnis zum Nutzen für den/die Patienten/in vertretbar und mit einem hohen Maß an Gesundheitsschutz und Sicherheit vereinbar sein (IVDR, Anhang I [1]). Unter Risiko versteht die IVDR die Kombination aus der *Wahrscheinlichkeit eines Schadenseintritts* und der *Schwere des Schadens* verstanden (Tab. 2).

**Figure 4:**
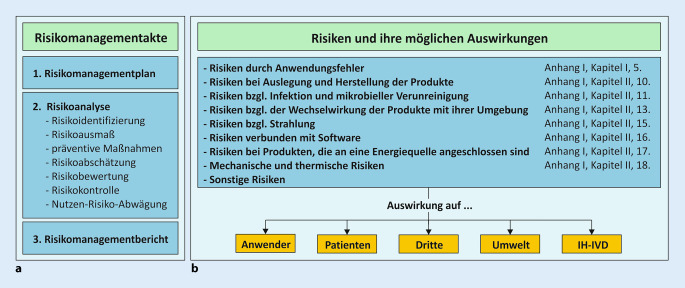

Für die Umsetzung des produktspezifischen Risikomanagements wurde eine neue Arbeitsanweisung für die QM-Dokumentation des IPH erstellt. Diese beschreibt die Erstellung einer produktspezifischen Risikomanagementakte (RM-Akte, Abb. 4a). Die RM-Akte enthält den geforderten Risikomanagementplan, die Risikoanalyse mit ihren Teilkomponenten und einen Risikomanagementbericht.

#### Risikomanagementplan

Für jedes IH-IVD muss ein Risikomanagementplan festgelegt und dokumentiert werden. Im IPH legen wir auf einem produktspezifischen Formular fest, welche „Risikokategorien“ für das zu betrachtende IH-IVD anzuwenden sind (Abb. 4b). Risiken, die aufgrund der Zweckbestimmung und der Herstellung auszuschließen sind, werden nicht weiter betrachtet. Die betrachteten Risikokategorien basieren auf den in Anhang I Kapitel II beschriebenen Sicherheitsanforderungen. Damit können viele der nach Anhang I zu erfüllenden Sicherheitsanforderungen betrachtet und in die Risikoanalyse einbezogen werden.

**Figure 5:**
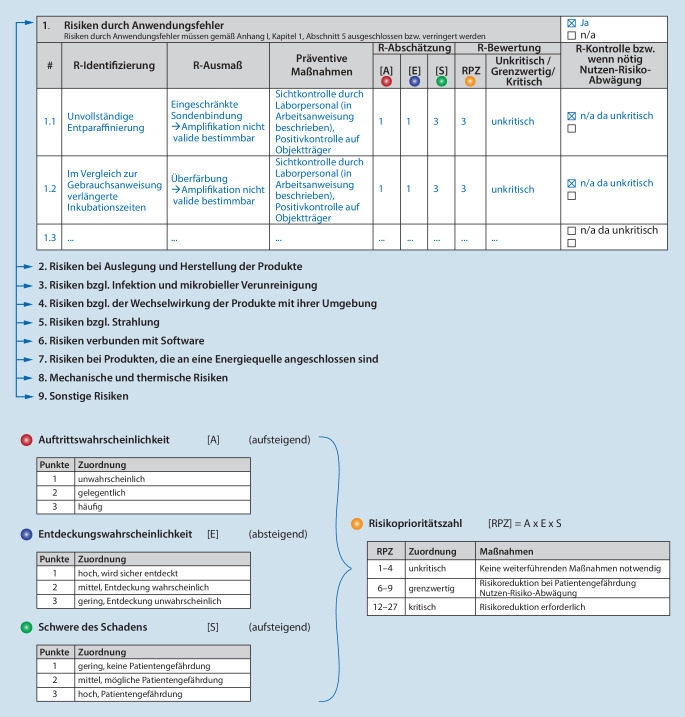

Die Verantwortlichkeiten im Risikomanagementprozess, die Bewertungsparameter für die Risikoabschätzung sowie die Kriterien für die Akzeptanz von Risiken sind in der Arbeitsanweisung zum produktspezifischen Risikomanagement festgelegt. Die Risikoabschätzung erfolgt anhand der Auftrittswahrscheinlichkeit (A), Entdeckungswahrscheinlichkeit (E) und der Schwere des Schadens (S). Die Risikobewertung erfolgt auf Grundlage der Risikoprioritätszahl (RPZ). Diese wird durch Multiplikation der Werte der Risikoabschätzung ermittelt (RPZ = [A] × [E] × [S], Abb. 5). Je höher die RPZ, desto höher ist das zugrunde liegende Risikopotential. Auf der Grundlage der resultierenden RPZ-Werte werden Maßnahmen festgelegt. Bei der Auswahl der geeignetsten Lösungen zur Risikominderung erfolgt gemäß Anhang I, Kapitel I, 4:

1. Das Risiko wird, wenn möglich, ausgeschlossen oder so weit wie möglich verringert;
2. ist das Risiko nicht auszuschließen, werden angemessene Schutzmaßnahmen ergriffen, einschließlich der Einrichtung von Alarmvorrichtungen (z. B. Kontrollen);
3. es werden Sicherheitsinformationen zu Restrisiken (z. B. Warn- und Vorsichtshinweise, Kontraindikationen) zur Verfügung gestellt; z. B. innerhalb von Arbeitsanweisungen oder Rezepturen. Diese werden in Anwendungsschulungen (z. B. im Rahmen der Einarbeitung) gezielt besprochen.

#### Risikoanalyse

Die Risikoanalyse wird von demjenigen Fachpersonal durchgeführt, das die größte Expertise für die Herstellung und Anwendung des zu betrachtenden IH-IVD hat (z. B. Laborpersonal, Naturwissenschaftler/in, Arzt/Ärztin, Bioinformatiker/in). Betrachtet werden potenzielle Risiken, sowohl für die Anwender/innen (z. B. Umgang mit Gefahrstoffen), für die Patient/innen (z. B. falsch-positive/-negative Ergebnisse), für mögliche Dritte (z. B. Exposition gegenüber Gefahrstoffen), für die Umwelt (z. B. Entsorgung) als auch für das Produkt selbst (z. B. Lagerbedingungen, Haltbarkeit; Abb. 4b).

Die Risikoanalyse umfasst folgende Komponenten und wird produktspezifisch und tabellarisch dokumentiert (Abb. 5):

- Risikoidentifikation: Beschreibung des Risikos und seiner Ursache;
- Risikoausmaß: Beschreibung der möglichen Folge oder des Schadens für Anwender/innen, für Patienten/innen, für Dritte, für die Umwelt oder für das Produkt;
- präventive Maßnahmen: Beschreibung der vorbeugenden Maßnahmen zur Risikominimierung;
- Risikoabschätzung: auf Grundlage der Auftrittswahrscheinlichkeit, Entdeckungswahrscheinlichkeit und der Schwere des Schadens;
- Risikobewertung: auf Grundlage der Risikoprioritätszahl (RPZ);
- Risikokontrolle/Nutzen-Risiko-Abwägung: ggf. Beschreibung weiterführender Maßnahmen und Kontrollmechanismen (z. B. Anwendungsschulung).

Ein mögliches Risiko im Zusammenhang mit der Herstellung und Anwendung muss im Verhältnis zum Nutzen für Patient/innen vertretbar und mit einem hohen Maß an Gesundheitsschutz und Sicherheit vereinbar sein (Anhang I, Kapitel I, 1.).

#### Risikomanagementbericht

Im Risikomanagementbericht wird bestätigt, dass ein mögliches Restrisiko bei der Herstellung und Anwendung im Verhältnis zum Nutzen für den/die Patienten/in kontrolliert, vertretbar und mit einem hohen Maß an Gesundheitsschutz und Sicherheit vereinbar ist. Sowohl das mit den einzelnen Gefährdungen verbundene Restrisiko als auch das Gesamtrisiko muss als akzeptabel eingestuft werden. Alle Dokumente des Risikomanagements stehen dem Anwenderkreis durch das QM-System zur Verfügung. Darüber hinaus werden sie über mögliche Restrisiken gesondert informiert (z. B. innerhalb der Arbeitsanweisung, im Rahmen von dokumentierten Schulungen oder protokollierten Besprechungen).

#### Risikoüberwachung

Das Risikomanagement muss ein kontinuierlicher Prozess sein, der regelmäßig und systematisch aktualisiert wird (Anhang I, Kapitel I, 3.). Mit Hilfe der im QM-System etablierten Analyse‑, Korrektur- und Verbesserungsprozesse (wie z. B. dem Fehler- und Maßnahmenmanagement, der regelmäßigen Durchführung von internen und externen Audits sowie der Teilnahme an Ringversuchen) werden mögliche Vorkommnisse im Zusammenhang mit IH-IVD erfasst und überprüft. Treten im Idealfall keine Vorkommnisse auf, wird die RM-Akte spätestens alle 2 Jahre auf Aktualität und Gültigkeit überprüft. Diese Überprüfung wird ebenfalls in der RM-Akte dokumentiert.

### Anhang I, Kapitel II: Anforderungen an Leistung, Auslegung und Herstellung

Kapitel II beschreibt die Anforderungen an Leistung, Auslegung und Herstellung von IVD. Die sichere Auslegung und Herstellung der IH-IVD wurde bereits in der RM-Akte betrachtet und bewertet. Die Leistung eines Produktes ist gemäß IVDR die Fähigkeit „seine vom Hersteller (Anmerkung: d. h. von den Pathologien) angegebene Zweckbestimmung zu erfüllen“ (Tab. 2). Durch die Analyse und Bewertung definierter Leistungsmerkmale werden die wissenschaftliche Validität, die Analyseleistung und ggf. die klinische Leistung vor der Anwendung der Produkte ermittelt und überprüft (= Leistungsbewertung, Tab. 2). Beispiele der zu betrachtenden Leistungsmerkmale sind in Kapitel II, 9.1. aufgeführt und werden auf der Grundlage der „vom Hersteller“ definierten produktspezifischen Zweckbestimmung festgelegt (s. unten und Abb. 3). Die Zweckbestimmung beeinflusst somit maßgeblich den Validierungsumfang und entscheidet, welche der in Kapitel II, 9.1. aufgeführten Leistungsmerkmale nachgewiesen werden müssen und welche ausgeschlossen werden können. Für die Leistungsbewertung von IH-Methoden zum Nachweis von Infektionserregern gibt ein Artikel der IVDR-Subgruppe der Ad-hoc-Kommission der Arbeitsgemeinschaft der Wissenschaftlichen Medizinischen Fachgesellschaften e. V (AWMF) Hilfestellung [14].

**Figure 6:**
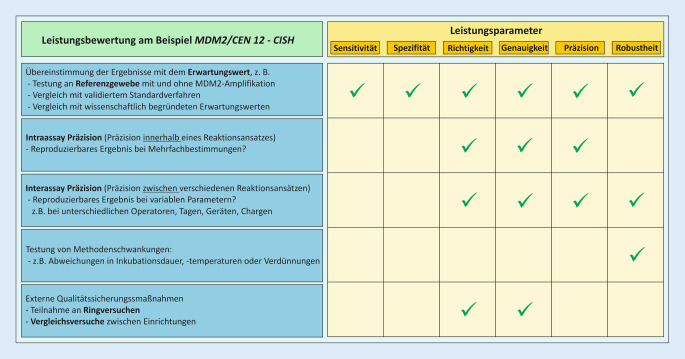

Die Methodenvalidierung am IPH orientiert sich an den Leitlinien des Sektorkomitees Pathologie/Neuropathologie, das für die Interpretation der Akkreditierungsanforderungen nach DIN EN ISO/IEC 17020 im Fachbereich Pathologie zuständig ist [2, 3]:

1. Definition der zu ermittelnden Leistungsmerkmale (Beispiel Abb. 6):
  Ausgehend von der Zweckbestimmung wird der Validierungsumfang festgelegt. Es wird geprüft welche, Leistungsmerkmale anwendbar sind. Die festgelegten Leistungsmerkmale werden auf einem validierungsspezifischen Formblatt dokumentiert.
2. Beschreibung des Untersuchungsverfahrens:
  Die Ergebnisse und Erkenntnisse aus der Entwicklungs- und Etablierungsphase fließen in die spezifische Arbeitsanweisung ein. Die Validierung wird entsprechend der Arbeitsanweisung durchgeführt.
3. Ermittlung der Leistungsmerkmale:
  Nachweis, dass die festgelegten Qualitätsanforderungen im konkreten Fall tatsächlich erfüllt werden.

Die abschließende Leistungsbewertung erfolgt im produktspezifischen Validierungsbericht. Dazu ist durch qualifiziertes Personal zu entscheiden, ob die Qualitätsanforderungen an das Verfahren erreicht wurden und es für die vorgesehenen Untersuchungen, gemäß der Zweckbestimmung, eingesetzt werden kann. Die kontinuierliche Überprüfung des validierten Verfahrens wird im laufenden Betrieb durch geeignete Kontrollmechanismen ständig überprüft und somit sichergestellt. Damit wird gewährleistet, dass das Verfahren reproduzierbar stabil und robust ist. Auch hier greift das etablierte QM-System mit den oben beschriebenen Analyse‑, Korrektur- und Verbesserungsprozessen.

### Anhang I, Kapitel III: Anforderungen an die mit dem Produkt gelieferten Informationen

In Kapitel III werden die Anforderungen an die mit dem Produkt gelieferten Informationen festgelegt. „Jedem Produkt werden die notwendigen Angaben beigefügt, die die Identifizierung des Produkts und des Herstellers ermöglichen, sowie alle für die Anwender oder gegebenenfalls Dritte relevanten Informationen über die Sicherheit und Leistung des Produkts“ (Anhang I, Kapitel III, 20.1.). Dies geschieht durch Kennzeichnungen und Gebrauchsanweisungen, für die Kapitel III Vorgaben macht. Dabei wird eine Gebrauchsanweisung wie folgt definiert: „Gebrauchsanweisung bezeichnet vom Hersteller zur Verfügung gestellte Informationen, in denen der Anwender über die Zweckbestimmung und korrekte Verwendung eines Produkts sowie über eventuell zu ergreifende Vorsichtsmaßnahmen unterrichtet wird“ (Tab. 2).

Gemäß den Anforderungen an IH-IVD nach Artikel 5 (5) ist die Pathologie sowohl Hersteller als auch Anwender zugleich, da die Produkte nicht abgegeben werden dürfen. Somit stehen dem Anwenderkreis jederzeit sämtliche relevanten Informationen über die Herstellung, Sicherheit und Leistung des IH-IVD zur Verfügung. Innerhalb des QM-Systems sind die Dokumente zur Zweckbestimmung, Risikoanalyse und Leistungsbewertung/Validierung für den Anwenderkreis jederzeit zugänglich. Darüber hinaus gibt es produkt- oder verfahrensspezifische Arbeitsanweisungen in der QM-Dokumentation. Diese sind entweder vom Anwenderkreis selbst erstellt oder die Anwender/innen sind im Rahmen des Schulungsprogramms darin geschult. Diese Arbeitsanweisungen enthalten wichtige Aspekte zur sicheren Anwendung der hausinternen Methoden und unterliegen wie das Risikomanagement einer regelmäßigen und systematischen Aktualisierung. Zur sicheren Anwendung von IH-IVD im Labor und damit notwendig zur Erfüllung der Sicherheitsanforderungen zählen auch einschlägige Informationen über Stoffe oder Gemische, die als gefährlich eingestuft sind, z. B. in Form von Sicherheitsdatenblättern und Betriebsanweisungen.

## Fazit/Diskussion

Für eine optimale Diagnostik und individuelle Therapiefindung werden in Instituten für Pathologie sowohl kommerzielle CE-IVD als auch IH-IVD gezielt eingesetzt [8]. Mit dem Erwägungsgrund 29 und dessen Umsetzung in Artikel 5 der IVDR erkennen die Verordnungsgeber den Nutzen und die Notwendigkeit von hausintern entwickelten Tests und ermöglichen deren Einsatz zur optimalen Patientenversorgung unter Bedingungen [8, 15]. Als Hersteller und zugleich Anwender von IH-IVD müssen Institute für Pathologie als Gesundheitseinrichtung dafür die Konformität mit Artikel 5 (5) der IVDR sicherstellen und die in Anhang I genannten Sicherheits- und Leistungsanforderungen erfüllen. Alle weiteren Anforderungen der IVDR gelten dann nicht für IH-IVD.

Im Januar 2023, wurde das Dokument MDCG-2023‑1 der *Medical Device Coordination Group* (MDCG) für Gesundheitseinrichtungen veröffentlicht, welches Leitlinien für einige Anforderungen aus Artikel 5 (5) enthält [13]. Die MDCG und ihre Aufgaben sind in den Artikeln 98 und 99 der IVDR beschrieben. Gemäß Artikel 99 c) hat sie die Aufgabe, Leitlinien für eine wirksame und harmonisierte Umsetzung der IVDR zu entwickeln, die jedoch rechtlich nicht bindend sind.

Für die praktische Umsetzung und für die inhaltliche Ausgestaltung von definitorischen Leerstellen und Unbestimmtheiten der Anforderungen engagieren sich viele Fachgesellschaften und Verbände und es haben sich neue Netzwerke gebildet, die ihr akademisches Fachwissen und ihre Expertise nutzen, um Hilfestellungen zu bieten. So unterstützen sie durch Handreichungen, Vorlagendokumente sowie durch Diskussionsveranstaltungen, Workshops und durch Erfahrungsaustausch. Die Ad-hoc-Kommission „*In-vitro-*Diagnostik“ der Arbeitsgemeinschaft der Wissenschaftlichen Medizinischen Fachgesellschaften e. V (AWMF) stellt auf ihrer Homepage zahlreiche Muster und Hilfestellungen (z. B. zum Risikomanagement und zur Leistungsbewertung) zur Verfügung [1]. Hilfreich sind auch die Empfehlungen des Bundesverbandes Deutscher Pathologen e. V. (BDP; [9–11]), die in Umsetzungsphasen die Implementierung der IVDR in der Pathologie beschreiben.

Solche Informationsquellen sind für die Institute ein wichtiger Schritt zur Umsetzung und Harmonisierung der Erfüllung der in Artikel 5 (5) genannten Anforderungen. Auch akademische Netzwerkstrukturen sind für die Interpretation der IVDR und deren praktische Umsetzung von Bedeutung.

**Figure 7:**
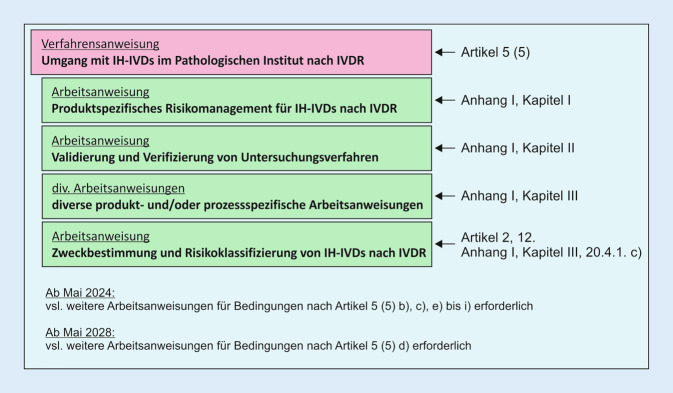

In Deutschland sind über 100 Institute für Pathologie oder Neuropathologie nach DIN EN ISO/IEC 17020 akkreditiert [7]. Die Implementierung der Anforderungen der IVDR für IH-IVD erfolgt somit in einer soliden, etablierten und unabhängig geprüften Qualitätsmanagementstruktur, in der viele Anforderungen bereits umgesetzt sind (z. B. die kontinuierliche Verifizierung der Methoden durch Qualitätskontrollen, Anwendungsschulungen oder das Fehlermanagement). Um dem Anhang I der IVDR gerecht zu werden, hat das Pathologische Institut in Heidelberg mehrere neue Anweisungsdokumente erstellt und in die bestehende DIN EN ISO/IEC 17020-konforme QM-Dokumentation integriert (Abb. 7). Teilweise konnte auch auf bereits bestehende QM-Dokumente zurückgegriffen werden, die jedoch an die Anforderungen des Anhangs I angepasst werden mussten.

Die intensive interne Auseinandersetzung mit den Anforderungen der IVDR (z. B. durch Gap-Analyse), die Erstellung der in Abb. 7 aufgeführten QM-Dokumente (Anweisungsdokumente und Formblätter), deren Implementierung in das bestehende QM-System, die dafür erforderlichen Schulungen und vor allem die in der IVDR vorgesehene produktspezifische Dokumentation durch die Expertise des Fachpersonals sind sehr ressourcenintensiv. Für die produktspezifische Dokumentation ist zunächst das IH-IVD zu definieren und einzugrenzen. Dies kann je nach Strategie für eine gesamte Prozesskette oder modular für einzelne Glieder bzw. Elemente geschehen und erfolgt in Form einer dokumentierten Zweckbestimmung. Auf der Grundlage dieser Festlegung werden das produktspezifische RM, das über das allgemeine RM im Rahmen einer Akkreditierung hinausgeht und die Leistungsbewertung durchgeführt und anschließend die Gebrauchsanweisungen in Form von Arbeitsanweisungen, dem QM-Handbuch entsprechend, erstellt. Eine neue Herausforderung stellen hier IH-Softwarelösungen dar, die durch die IVDR nun auch unter die Definition eines IVD fallen.

Für eine universitäre Einrichtung, wie das IPH, stellt die Sicherstellung der IVDR-Konformität – neben den Kernaufgaben in der Krankenversorgung, neben Lehre und Forschung, neben der Weiterentwicklung von Methoden für eine optimale und zielgerichtete personalisierte Diagnostik und neben der Aufrechterhaltung des sich stetig weiterentwickelnden QM-Systems – eine zusätzliche große personelle und zeitliche Herausforderung dar.

## Fazit für die Praxis

- Seit Mai 2022 müssen Gesundheitseinrichtungen den Anhang I der Verordnung (EU) 2017/746 (IVDR) für *In-house*-*in-vitro*-Diagnostika (IH-IVD) erfüllen.
- Eine Akkreditierung nach DIN EN ISO/IEC 17020 ist nicht erforderlich, sie bildet aber eine solide Basis für die Konformität mit Artikel 5 (5) der IVDR.
- Durchführung einer Lücken-(Gap‑)Analyse sinnvoll → Artikel 5 (5) *vs*. etabliertes Qualitätsmanagement (QM)-System → IVDR-Anforderungen, die noch nicht abgebildet sind, implementieren.
- Bestehende etablierte QM-Strukturen können und sollten genutzt und bei Bedarf erweitert werden.
- Hilfestellungen, Muster, Vorlagen und Checklisten helfen bei der Umsetzung der Bedingungen für IH-IVD.
- Mit dem Ziel einer harmonisierten Umsetzung arbeiten verschiedene akademische Netzwerkstrukturen, Verbände und Fachgesellschaften an der Entwicklung und Veröffentlichung von Hilfestellungen (z. B. AWMF [Arbeitsgemeinschaft der Wissenschaftlichen Medizinischen Fachgesellschaften e. V], BDP [Bundesverband Deutscher Pathologen e. V.]).

## References

[1] Arbeitsgemeinschaft der Wissenschaftlichen Medizinischen Fachgesellschaften e. V. (AWMF) Handreichungen und Muster der Ad hoc Kommission In vitro Diagnostika. https://www.awmf.org/service/arbeitshilfen-und-formulare#c1797. Zugegriffen: 25. Mai 2023

[2] DAkkS (2016). Leitfaden des Sektorkomitees Pathologie/Neuropathologie für die Validierung von Untersuchungsverfahren in der Immunhistologie - Kennung: 71 SD 4 028.

[3] DAkkS (2016). Leitfaden des Sektorkomitees Pathologie/Neuropathologie für die Validierung von Untersuchungsverfahren in der Molekularpathologie - Kennung: 71 SD 4 037.

[4] DakkS Medizinische Diagnostik - Fachbereich 3.5 im Überblick. https://www.dakks.de/de/fb-3.5.html. Zugegriffen: 25. Mai 2023

[5] EUDAMED - Europäische Datenbank für Medizinprodukte. https://ec.europa.eu/tools/eudamed/#/screen/home. Zugegriffen: 25. Mai 2023

[6] Europäische Kommission Nando (New Approach Notified and Designated Organisations) Information System. https://ec.europa.eu/growth/tools-databases/nando/. Zugegriffen: 25. Mai 2022

[7] Holl-Ulrich K, Hagel C, Köhler G (2022). Akkreditierung in der Pathologie und Neuropathologie. Pathologie.

[8] Kahles A, Goldschmid H, Volckmar AL (2022). Struktur und Inhalt der EU-IVDR. Pathologie.

[9] Kazmierczak M (2022) Empfehlungen zur IVDR des Bundesverband Deutscher Pathologen e. V. Einleitung. https://www.pathologie.de/?eID=downloadtool&uid=2233. Zugegriffen: 25. Mai 2023

[10] Kazmierczak M (2022) Empfehlungen zur IVDR des Bundesverband Deutscher Pathologen e. V. Phase 1 – Inventur durchfuhren. https://www.pathologie.de/?eID=downloadtool&uid=2234. Zugegriffen: 25. Mai 2023

[11] Kazmierczak M (2022) Empfehlungen zur IVDR des Bundesverband Deutscher Pathologen e. V. Phase 2 – Sicherheits- und Leistungsnachweis. https://www.pathologie.de/?eID=downloadtool&uid=2239. Zugegriffen: 25. Mai 2023

[12] Medical Device Coordination Group (2023) MDCG 2020-16 rev.2 - Guidance on Classification Rules for in vitro Diagnostic Medical Devices under Regulation (EU) 2017/746. https://health.ec.europa.eu/system/files/2023-02/md_mdcg_2020_guidance_classification_ivd-md_en.pdf. Zugegriffen: 25. Mai 2023

[13] Medical Device Coordination Group (2023) MDCG 2023‑1 - Guidance on the health institution exception under Article 5(5) of Regulation (EU) 2017/745 and Regulation (EU) 2017/746. https://health.ec.europa.eu/system/files/2023-01/mdcg_2023-1_en.pdf. Zugegriffen: 25. Mai 2023

[14] Rabenau HF, Hofmann J, Hunfeld KP, Reischl U, Schubert A, Spitzenberger F, IVDR-Subgruppe der Ad-Hoc-Kommission IVD der Arbeitsgemeinschaft der Wissenschaftlichen Medizinischen Fachgesellschaften e. V. (AWMF) (2022). Die neue In-vitro-Diagnostika-Verordnung (IVDR): Hilfestellung bei der Validierung/Verifizierung von im diagnostischen Laboratorium eingesetzten bzw. entwickelten und angewendeten Methoden zum Nachweis von Infektionserregern. GMS Z Forder Qual Med Lab.

[15] Stenzinger A, Weichert W (2020). Einfluss der neuen In-vitro-Diagnostik-Regulation (IVDR) der Europäischen Union auf die Pathologie. Was ist wichtig?. Pathologe.

[16] Verordnung (EU) 2017/746 des Europäischen Parlaments und des Rates vom 5. April 2017 über In-vitro-Diagnostika (IVDR). Amtsblatt der Europäischen Union L 117 vom 05.05.2017, S. 176.

[17] Verordnung (EU) 2022/112 des Europäischen Parlaments und des Rates vom 25. Januar 2022 zur Änderung der Verordnung (EU) 2017/746 hinsichtlich der Übergangsbestimmungen für bestimmte In-vitro-Diagnostika und des späteren Geltungsbeginns der Bedingungen für hausinterne Produkte. Amtsblatt der Europäischen Union L 19 vom 28.01.2022, S. 3.

